# Face Recognition Systems: A Survey

**DOI:** 10.3390/s20020342

**Published:** 2020-01-07

**Authors:** Yassin Kortli, Maher Jridi, Ayman Al Falou, Mohamed Atri

**Affiliations:** 1 AI-ED Department, Yncrea Ouest, 20 rue du Cuirassé de Bretagne, 29200 Brest, France; maher.jridi@isen-ouest.yncrea.fr (M.J.); ayman.alfalou@isen-ouest.yncrea.fr (A.A.F.); 2 Electronic and Micro-electronic Laboratory, Faculty of Sciences of Monastir, University of Monastir, Monastir 5000, Tunisia; 3 College of Computer Science, King Khalid University, Abha 61421, Saudi Arabia; matri@kku.edu.sa

**Keywords:** face recognition systems, person identification, biometric systems, survey

## Abstract

Over the past few decades, interest in theories and algorithms for face recognition has been growing rapidly. Video surveillance, criminal identification, building access control, and unmanned and autonomous vehicles are just a few examples of concrete applications that are gaining attraction among industries. Various techniques are being developed including local, holistic, and hybrid approaches, which provide a face image description using only a few face image features or the whole facial features. The main contribution of this survey is to review some well-known techniques for each approach and to give the taxonomy of their categories. In the paper, a detailed comparison between these techniques is exposed by listing the advantages and the disadvantages of their schemes in terms of robustness, accuracy, complexity, and discrimination. One interesting feature mentioned in the paper is about the database used for face recognition. An overview of the most commonly used databases, including those of supervised and unsupervised learning, is given. Numerical results of the most interesting techniques are given along with the context of experiments and challenges handled by these techniques. Finally, a solid discussion is given in the paper about future directions in terms of techniques to be used for face recognition.

## 1. Introduction

The objective of developing biometric applications, such as facial recognition, has recently become important in smart cities. In addition, many scientists and engineers around the world have focused on establishing increasingly robust and accurate algorithms and methods for these types of systems and their application in everyday life. All types of security systems must protect all personal data. The most commonly used type for recognition is the password. However, through the development of information technologies and security algorithms, many systems are beginning to use many biometric factors for recognition task [1,2,3,4]. These biometric factors make it possible to identify people’s identity by their physiological or behavioral characteristics. They also provide several advantages, for example, the presence of a person in front of the sensor is sufficient, and there is no more need to remember several passwords or confidential codes anymore. In this context, many recognition systems based on different biometric factors such as iris, fingerprints [5], voice [6], and face have been deployed in recent years.

Systems that identify people based on their biological characteristics are very attractive because they are easy to use. The human face is composed of different structures and characteristics. For this reason, in recent years, it has become one of the most widely used biometric authentication systems, given its potential in many applications and fields (surveillance, home security, border control, and so on) [7,8,9]. Facial recognition system as an ID (identity) is already being offered to consumers outside of phones, including at airport check-ins, sports stadiums, and concerts. In addition, this system does not require the intervention of people to operate, which makes it possible to identify people only from images obtained from the camera. In addition, many biometric systems that are developed using different types of search provide good identification accuracy. However, it would be interesting to develop new biometric systems for face recognition in order to reach real-time constraints.

Owing to the huge volume of data generated and rapid advancement in artificial intelligence techniques, traditional computing models have become inadequate to process data, especially for complex applications like those related to feature extraction. Graphics processing units (GPUs) [4], central processing unit (CPU) [3], and programmable gate arrays (FPGAs) [10] are required to efficiently perform complex computing tasks. GPUs have computing cores that are several orders of magnitude larger than traditional CPU and allow greater capacity to perform parallel computing. Unlike GPUs, the FPGAs have a flexible hardware configuration and offer better performance than GPUs in terms of energy efficiency. However, FPGAs present a major drawback related to the programming time, which is higher than that of CPU and GPU.

There are many computer vision approaches proposed to address face detection or recognition tasks with high robustness and discrimination, such as local, subspace, and hybrid approaches [10,11,12,13,14,15,16]. However, several issues still need to be addressed owing to various challenges, such as head orientation, lighting conditions, and facial expression. The most interesting techniques are developed to face all these challenges, and thus develop reliable face recognition systems. Nevertheless, they require high processing time, high memory consumption, and are relatively complex.

Rapid advances in technologies such as digital cameras, portable devices, and increased demand for security make the face recognition system one of the primary biometric technologies.

To sum up, the contributions of this paper review are as follows:

1. We first introduced face recognition as a biometric technique.
2. We presented the state of the art of the existing face recognition techniques classified into three approaches: local, holistic, and hybrid.
3. The surveyed approaches were summarized and compared under different conditions.
4. We presented the most popular face databases used to test these approaches.
5. We highlighted some new promising research directions.

## 2. Face Recognition Systems Survey

### 2.1. Essential Steps of Face Recognition Systems

Before detailing the techniques used, it is necessary to make a brief description of the problems that must be faced and solved in order to perform the face recognition task correctly. For several security applications, as detailed in the works of [17,18,19,20,21,22], the characteristics that make a face recognition system useful are the following: its ability to work with both videos and images, to process in real time, to be robust in different lighting conditions, to be independent of the person (regardless of hair, ethnicity, or gender), and to be able to work with faces from different angles. Different types of sensors, including RGB, depth, EEG, thermal, and wearable inertial sensors, are used to obtain data. These sensors may provide extra information and help the face recognition systems to identify face images in both static images and video sequences. Moreover, three categories of sensors that may improve the reliability and the accuracy of a face recognition system by tackling the challenges include illumination variation, head pose, and facial expression in pure image/video processing. The first group is non-visual sensors, such as audio, depth, and EEG sensors, which provide extra information in addition to the visual dimension and improve the recognition reliability, for example, in illumination variation and position shift situation. The second is detailed-face sensors, which detect a small dynamic change of a face component, such as eye-trackers, which may help differentiate the background noise and the face images. The last is target-focused sensors, such as infrared thermal sensors, which can facilitate the face recognition systems to filter useless visual contents and may help resistance illumination variation.

**Figure 1:**
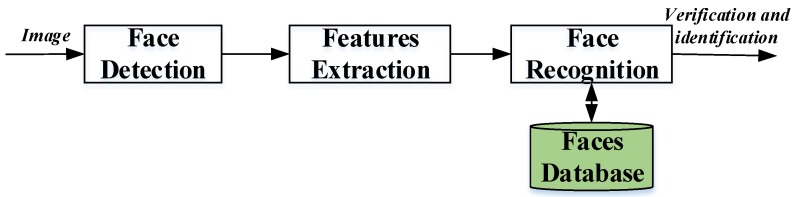
Face recognition structure [3,23].

Three basic steps are used to develop a robust face recognition system: (1) face detection, (2) feature extraction, and (3) face recognition (shown in Figure 1) [3,23]. The face detection step is used to detect and locate the human face image obtained by the system. The feature extraction step is employed to extract the feature vectors for any human face located in the first step. Finally, the face recognition step includes the features extracted from the human face in order to compare it with all template face databases to decide the human face identity.

- *Face Detection*: The face recognition system begins first with the localization of the human faces in a particular image. The purpose of this step is to determine if the input image contains human faces or not. The variations of illumination and facial expression can prevent proper face detection. In order to facilitate the design of a further face recognition system and make it more robust, pre-processing steps are performed. Many techniques are used to detect and locate the human face image, for example, Viola–Jones detector [24,25], histogram of oriented gradient (HOG) [13,26], and principal component analysis (PCA) [27,28]. Also, the face detection step can be used for video and image classification, object detection [29], region-of-interest detection [30], and so on.
- *Feature Extraction*: The main function of this step is to extract the features of the face images detected in the detection step. This step represents a face with a set of features vector called a “signature” that describes the prominent features of the face image such as mouth, nose, and eyes with their geometry distribution [31,32]. Each face is characterized by its structure, size, and shape, which allow it to be identified. Several techniques involve extracting the shape of the mouth, eyes, or nose to identify the face using the size and distance [3]. HOG [33], Eigenface [34], independent component analysis (ICA), linear discriminant analysis (LDA) [27,35], scale-invariant feature transform (SIFT) [23], gabor filter, local phase quantization (LPQ) [36], Haar wavelets, Fourier transforms [31], and local binary pattern (LBP) [3,10] techniques are widely used to extract the face features.
- *Face Recognition*: This step considers the features extracted from the background during the feature extraction step and compares it with known faces stored in a specific database. There are two general applications of face recognition, one is called identification and another one is called verification. During the identification step, a test face is compared with a set of faces aiming to find the most likely match. During the identification step, a test face is compared with a known face in the database in order to make the acceptance or rejection decision [7,19]. Correlation filters (CFs) [18,37,38], convolutional neural network (CNN) [39], and also k-nearest neighbor (K-NN) [40] are known to effectively address this task.

### 2.2. Classification of Face Recognition Systems

Compared with other biometric systems such as the eye, iris, or fingerprint recognition systems, the face recognition system is not the most efficient and reliable [5]. Moreover, this biometric system has many constraints resulting from many challenges, despite all the above advantages. The recognition under the controlled environments has been saturated. Nevertheless, in uncontrolled environments, the problem remains open owing to large variations in lighting conditions, facial expressions, age, dynamic background, and so on. In this paper survey, we review the most advanced face recognition techniques proposed in controlled/uncontrolled environments using different databases.

**Figure 2:**
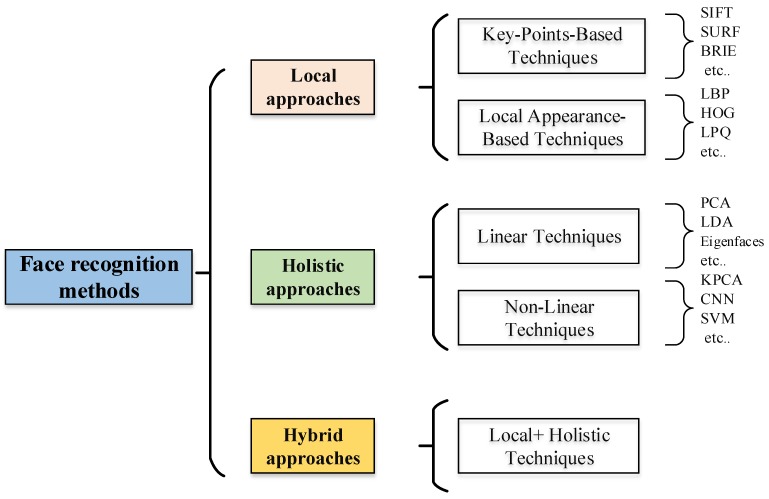
Face recognition methods. SIFT, scale-invariant feature transform; SURF, scale-invariant feature transform; BRIEF, binary robust independent elementary features; LBP, local binary pattern; HOG, histogram of oriented gradients; LPQ, local phase quantization; PCA, principal component analysis; LDA, linear discriminant analysis; KPCA, kernel PCA; CNN, convolutional neural network; SVM, support vector machine.

Several systems are implemented to identify a human face in 2D or 3D images. In this review paper, we will classify these systems into three approaches based on their detection and recognition method (Figure 2): (1) local, (2) holistic (subspace), and (3) hybrid approaches. The first approach is classified according to certain facial features, not considering the whole face. The second approach employs the entire face as input data and then projects into a small subspace or in correlation plane. The third approach uses local and global features in order to improve face recognition accuracy.

## 3. Local Approaches

In the context of face recognition, local approaches treat only some facial features. They are more sensitive to facial expressions, occlusions, and pose [1]. The main objective of these approaches is to discover distinctive features. Generally, these approaches can be divided into two categories: (1) local appearance-based techniques are used to extract local features, while the face image is divided into small regions (patches) [3,32]. (2) Key-points-based techniques are used to detect the points of interest in the face image, after which the features localized on these points are extracted.

### 3.1. Local Appearance-Based Techniques

It is a geometrical technique, also called feature or analytic technique. In this case, the face image is represented by a set of distinctive vectors with low dimensions or small regions (patches). Local appearance-based techniques focus on critical points of the face such as the nose, mouth, and eyes to generate more details. Also, it takes into account the particularity of the face as a natural form to identify and use a reduced number of parameters. In addition, these techniques describe the local features through pixel orientations, histograms [13,26], geometric properties, and correlation planes [3,33,41].

**Figure 3:**
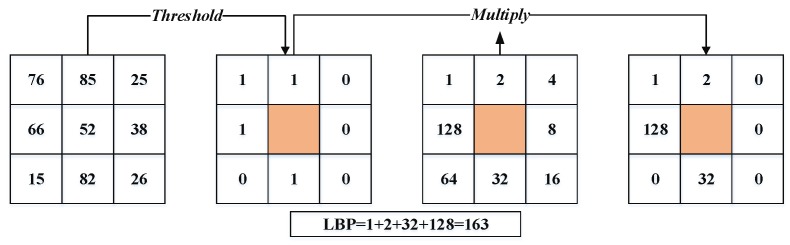
The local binary pattern (LBP) descriptor [19].

**Figure 4:**
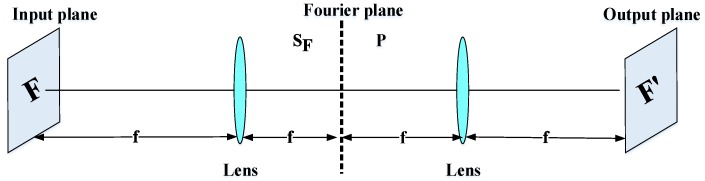
All “4f” optical configuration [37].

**Figure 5:**
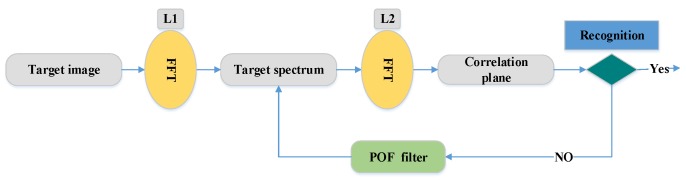
Flowchart of the VanderLugt correlator (VLC) technique [4]. FFT, fast Fourier transform; POF, phase-only filter.

**Figure 6:**
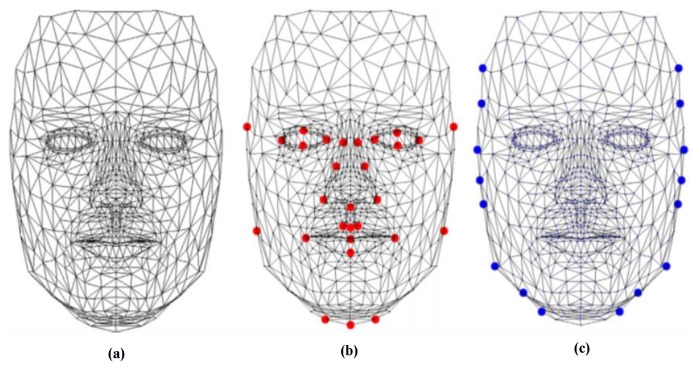
(**a**) Creation of the 3D face of a person, (**b**) results of the detection of 29 landmarks of a face using the active shape model, (**c**) results of the detection of 26 landmarks of a face [3].

- Local binary pattern (LBP) and it’s variant: LBP is a great general texture technique used to extract features from any object [16]. It has widely performed in many applications such as face recognition [3], facial expression recognition, texture segmentation, and texture classification. The LBP technique first divides the facial image into spatial arrays. Next, within each array square, a 3×3 pixel matrix (p1……p8) is mapped across the square. The pixel of this matrix is a threshold with the value of the center pixel $\left(p_{0}\right)$ (i.e., use the intensity value of the center pixel $i\left(p_{0}\right)$ as a reference for thresholding) to produce the binary code. If a neighbor pixel’s value is lower than the center pixel value, it is given a zero; otherwise, it is given one. The binary code contains information about the local texture. Finally, for each array square, a histogram of these codes is built, and the histograms are concatenated to form the feature vector. The LBP is defined in a matrix of size 3 × 3, as shown in Equation (1).
  (1) $$\mathrm{LBP}=\overset{8}{\underset{p=1}{\sum }}2^{p}s\left(i_{0}-i_{p}\right),\;with\;s\left(x\right)=\{\begin{array}{cc} 1 & x\geq 0\\ 0 & x<0 \end{array},$$
  where $i_{0}$ and $i_{p}$ are the intensity value of the center pixel and neighborhood pixels, respectively. Figure 3 illustrates the procedure of the LBP technique.
  Khoi et al. [20] propose a fast face recognition system based on LBP, pyramid of local binary pattern (PLBP), and rotation invariant local binary pattern (RI-LBP). Xi et al. [15] have introduced a new unsupervised deep learning-based technique, called local binary pattern network (LBPNet), to extract hierarchical representations of data. The LBPNet maintains the same topology as the convolutional neural network (CNN). The experimental results obtained using the public benchmarks (i.e., LFW and FERET) have shown that LBPNet is comparable to other unsupervised techniques. Laure et al. [40] have implemented a method that helps to solve face recognition issues with large variations of parameters such as expression, illumination, and different poses. This method is based on two techniques: LBP and K-NN techniques. Owing to its invariance to the rotation of the target image, LBP become one of the important techniques used for face recognition. Bonnen et al. [42] proposed a variant of the LBP technique named “multiscale local binary pattern (MLBP)” for features’ extraction. Another LBP extension is the local ternary pattern (LTP) technique [43], which is less sensitive to the noise than the original LBP technique. This technique uses three steps to compute the differences between the neighboring ones and the central pixel. Hussain et al. [36] develop a local quantized pattern (LQP) technique for face representation. LQP is a generalization of local pattern features and is intrinsically robust to illumination conditions. The LQP features use the disk layout to sample pixels from the local neighborhood and obtain a pair of binary codes using ternary split coding. These codes are quantized, with each one using a separately learned codebook.
- Histogram of oriented gradients (HOG) [44]: The HOG is one of the best descriptors used for shape and edge description. The HOG technique can describe the face shape using the distribution of edge direction or light intensity gradient. The process of this technique done by sharing the whole face image into cells (small region or area); a histogram of pixel edge direction or direction gradients is generated of each cell; and, finally, the histograms of the whole cells are combined to extract the feature of the face image. The feature vector computation by the HOG descriptor proceeds as follows [10,13,26,45]: firstly, divide the local image into regions called cells, and then calculate the amplitude of the first-order gradients of each cell in both the horizontal and vertical direction. The most common method is to apply a 1D mask, [–1 0 1].
  (2) $$G_{x}\left(x,\;y\right)=I\left(x+1,\;y\right)-I\left(x-1,\;y\right),$$
  (3) $$G_{y}\left(x,\;y\right)=I\left(x,\;y+1\right)-I\left(x,\;y-1\right),$$
  where I(x, y) is the pixel value of the point (x, y) and $G_{x}\left(x,\;y\right)$ and $G_{y}\left(x,\;y\right)$ denote the horizontal gradient amplitude and the vertical gradient amplitude, respectively. The magnitude of the gradient and the orientation of each pixel (*x*, *y*) are computed as follows:
  (4) $$G\left(x,\;y\right)=\sqrt{G_{x}{\left(x,\;y\right)}^{2}+G_{y}{\left(x,\;y\right)}^{2}},$$
  (5) $$\theta \left(x,\;y\right)=\tan^{-1}\left(\frac{G_{y}\left(x,\;y\right)}{G_{x}\left(x,\;y\right)}\right).$$
  The magnitude of the gradient and the orientation of each pixel in the cell are voted in nine bins with the tri-linear interpolation. The histograms of each cell are generated pixel based on direction gradients and, finally, the histograms of the whole cells are combined to extract the feature of the face image. Karaaba et al. [44] proposed a combination of different histograms of oriented gradients (HOG) to perform a robust face recognition system. This technique is named “multi-HOG”.
  The authors create a vector of distances between the target and the reference face images for identification. Arigbabu et al. [46] proposed a novel face recognition system based on the Laplacian filter and the pyramid histogram of gradient (PHOG) descriptor. In addition, to investigate the face recognition problem, support vector machine (SVM) is used with different kernel functions.
- Correlation filters: Face recognition systems based on the correlation filter (CF) have given good results in terms of robustness, location accuracy, efficiency, and discrimination. In the field of facial recognition, the correlation techniques have attracted great interest since the first use of an optical correlator [47]. These techniques provide the following advantages: high ability for discrimination, desired noise robustness, shift-invariance, and inherent parallelism. On the basis of these advantages, many optoelectronic hybrid solutions of correlation filters (CFs) have been introduced such as the joint transform correlator (JTC) [48] and VanderLugt correlator (VLC) [47] techniques. The purpose of these techniques is to calculate the degree of similarity between target and reference images. The decision is taken by the detection of a correlation peak. Both techniques (VLC and JTC) are based on the “4f ” optical configuration [37]. This configuration is created by two convergent lenses (Figure 4). The face image F is processed by the fast Fourier transform (FFT) based on the first lens in the Fourier plane $S_{F}$. In this Fourier plane, a specific filter P is applied (for example, the phase-only filter (POF) filter [2]) using optoelectronic interfaces. Finally, to obtain the filtered face image $F^{\prime }$ (or the correlation plane), the inverse FFT (IFFT) is made with the second lens in the output plane.
  For example, the VLC technique is done by two cascade Fourier transform structures realized by two lenses [4], as presented in Figure 5. The VLC technique is presented as follows: firstly, a 2D-FFT is applied to the target image to get a target spectrum S. After that, a multiplication between the target spectrum and the filter obtain with the 2D-FFT of a reference image is affected, and this result is placed in the Fourier plane. Next, it provides the correlation result recorded on the correlation plane, where this multiplication is affected by inverse FF.
  The correlation result, described by the peak intensity, is used to determine the similarity degree between the target and reference images.
  (6) $$C=FFT^{-1}\left\{S^{*}\circ POF\right\},$$
  where $FFT^{-1}$ stands for the inverse fast FT (FFT) operation, * represents the conjugate operation, and ∘ denotes the element-wise array multiplication. To enhance the matching process, Horner and Gianino [49] proposed a phase-only filter (POF). The POF filter can produce correlation peaks marked with enhanced discrimination capability. The POF is an optimized filter defined as follows:
  (7) $$H_{POF}\left(u,v\right)=\frac{S^{*}\left(u,v\right)}{\left|S\left(u,v\right)\right|},$$
  where $S^{*}\left(u,v\right)$ is the complex conjugate of the 2D-FFT of the reference image. To evaluate the decision, the peak to correlation energy (PCE) is defined as the energy in the correlation peaks’ intensity normalized to the overall energy of the correlation plane.
  (8) $$PCE=\frac{\sum_{i,j}^{N}E_{peak}\left(i,j\right)}{\sum_{i,j}^{M}E_{correlation-plane}\left(i,j\right)},$$
  where i, j are the coefficient coordinates; M and N are the size of the correlation plane and the size of the peak correlation spot, respectively; $E_{peak}$ is the energy in the correlation peaks; and $E_{correlation-plane}$ is the overall energy of the correlation plane. Correlation techniques are widely applied in recognition and identification applications [4,37,50,51,52,53]. For example, in the work of [4], the authors presented the efficiency performances of the VLC technique based on the “4f” configuration for identification using GPU Nvidia Geforce 8400 GS. The POF filter is used for the decision. Another important work in this area of research is presented by Leonard et al. [50], which presented good performance and the simplicity of the correlation filters for the field of face recognition. In addition, many specific filters such as POF, BPOF, Ad, IF, and so on are used to select the best filter based on its sensitivity to the rotation, scale, and noise. Napoléon et al. [3] introduced a novel system for identification and verification fields based on an optimized 3D modeling under different illumination conditions, which allows reconstructing faces in different poses. In particular, to deform the synthetic model, an active shape model for detecting a set of key points on the face is proposed in Figure 6. The VanderLugt correlator is proposed to perform the identification and the LBP descriptor is used to optimize the performances of a correlation technique under different illumination conditions. The experiments are performed on the Pointing Head Pose Image Database (PHPID) database with an elevation ranging from −30° to +30°.

### 3.2. Key-Points-Based Techniques

The key-points-based techniques are used to detect specific geometric features, according to some geometric information of the face surface (e.g., the distance between the eyes, the width of the head). These techniques can be defined by two significant steps, key-point detection and feature extraction [3,30,54,55]. The first step focuses on the performance of the detectors of the key-point features of the face image. The second step focuses on the representation of the information carried with the key-point features of the face image. Although these techniques can solve the missing parts and occlusions, scale invariant feature transform (SIFT), binary robust independent elementary features (BRIEF), and speeded-up robust features (SURF) techniques are widely used to describe the feature of the face image.

**Figure 7:**
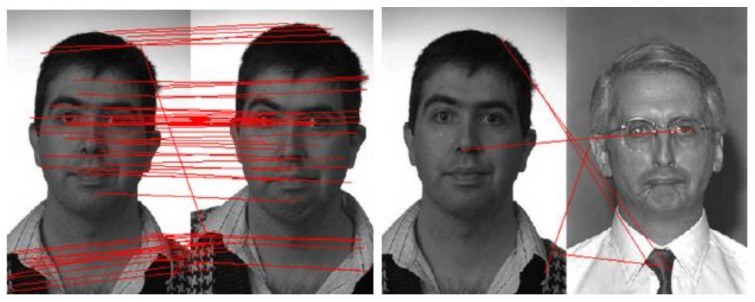
Face recognition based on the speeded-up robust features (SURF) descriptor [58]: recognition using fast library for approximate nearest neighbors (FLANN) distance.

**Figure 8:**
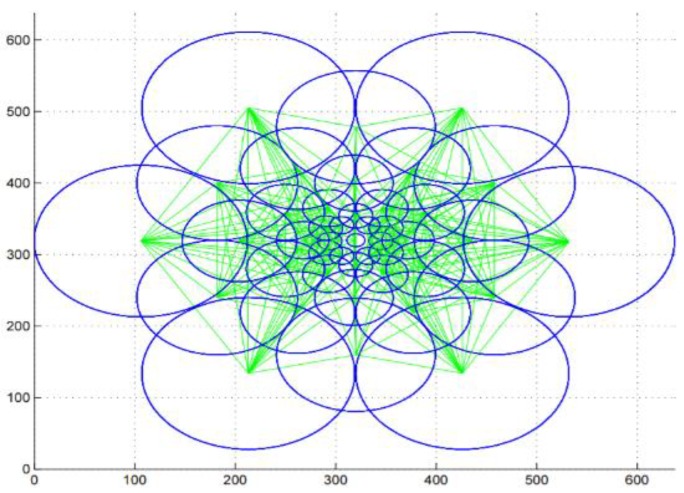
Fast retina keypoint (FREAK) descriptor used 43 sampling patterns [19].

- Scale invariant feature transform (SIFT) [56,57]: SIFT is an algorithm used to detect and describe the local features of an image. This algorithm is widely used to link two images by their local descriptors, which contain information to make a match between them. The main idea of the SIFT descriptor is to convert the image into a representation composed of points of interest. These points contain the characteristic information of the face image. SIFT presents invariance to scale and rotation. It is commonly used today and fast, which is essential in real-time applications, but one of its disadvantages is the time of matching of the critical points. The algorithm is realized in four steps: (1) detection of the maximum and minimum points in the space-scale, (2) location of characteristic points, (3) assignment of orientation, and (4) a descriptor of the characteristic point. A framework to detect the key-points based on the SIFT descriptor was proposed by L. Lenc et al. [56], where they use the SIFT technique in combination with a Kepenekci approach for the face recognition.
- Speeded-up robust features (SURF) [29,57]: the SURF technique is inspired by SIFT, but uses wavelets and an approximation of the Hessian determinant to achieve better performance [29]. SURF is a detector and descriptor that claims to achieve the same, or even better, results in terms of repeatability, distinction, and robustness compared with the SIFT descriptor. The main advantage of SURF is the execution time, which is less than that used by the SIFT descriptor. Besides, the SIFT descriptor is more adapted to describe faces affected by illumination conditions, scaling, translation, and rotation [57]. To detect feature points, SURF seeks to find the maximum of an approximation of the Hessian matrix using integral images to dramatically reduce the processing computational time. Figure 7 shows an example of SURF descriptor for face recognition using AR face datasets [58].
- Binary robust independent elementary features (BRIEF) [30,57]: BRIEF is a binary descriptor that is simple and fast to compute. This descriptor is based on the differences between the pixel intensity that are similar to the family of binary descriptors such as binary robust invariant scalable (BRISK) and fast retina keypoint (FREAK) in terms of evaluation. To reduce noise, the BRIEF descriptor smoothens the image patches. After that, the differences between the pixel intensity are used to represent the descriptor. This descriptor has achieved the best performance and accuracy in pattern recognition.
- Fast retina keypoint (FREAK) [57,59]: the FREAK descriptor proposed by Alahi et al. [59] uses a retinal sampling circular grid. This descriptor uses 43 sampling patterns based on retinal receptive fields that are shown in Figure 8. To extract a binary descriptor, these 43 receptive fields are sampled by decreasing factors as the distance from the thousand potential pairs to a patch’s center yields. Each pair is smoothed with Gaussian functions. Finally, the binary descriptors are represented by setting a threshold and considering the sign of differences between pairs.

### 3.3. Summary of Local Approaches

Table 1 summarizes the local approaches that we presented in this section. Various techniques are introduced to locate and to identify the human faces based on some regions of the face, geometric features, and facial expressions. These techniques provide robust recognition under different illumination conditions and facial expressions. Furthermore, they are sensitive to noise, and invariant to translations and rotations.

## 4. Holistic Approach

Holistic or subspace approaches are supposed to process the whole face, that is, they do not require extracting face regions or features points (eyes, mouth, noses, and so on). The main function of these approaches is to represent the face image by a matrix of pixels, and this matrix is often converted into feature vectors to facilitate their treatment. After that, these feature vectors are implemented in low dimensional space. However, holistic or subspace techniques are sensitive to variations (facial expressions, illumination, and poses), and these advantages make these approaches widely used. Moreover, these approaches can be divided into categories, including linear and non-linear techniques, based on the method used to represent the subspace.

### 4.1. Linear Techniques

The most popular linear techniques used for face recognition systems are Eigenfaces (principal component analysis; PCA) technique, Fisherfaces (linear discriminative analysis; LDA) technique, and independent component analysis (ICA).

**Figure 9:**
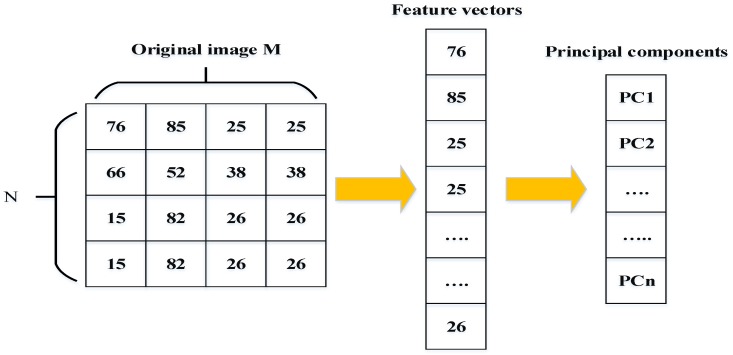
Example of dimensional reduction when applying principal component analysis (PCA) [62].

**Figure 10:**
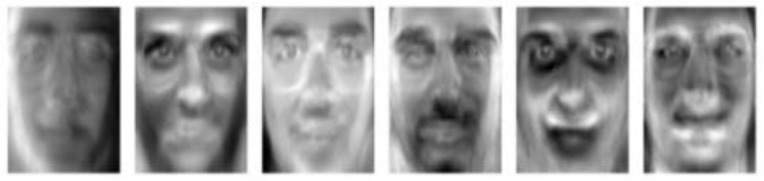
The first five Eigenfaces built from the ORL database [63].

**Figure 11:**
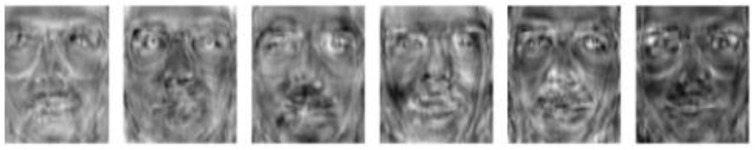
The first five Fisherfaces obtained from the ORL database [63].

- Eigenface [34] and principal component analysis (PCA) [27,62]: Eigenfaces is one of the popular methods of holistic approaches used to extract features points of the face image. This approach is based on the principal component analysis (PCA) technique. The principal components created by the PCA technique are used as Eigenfaces or face templates. The PCA technique transforms a number of possibly correlated variables into a small number of incorrect variables called “principal components”. The purpose of PCA is to reduce the large dimensionality of the data space (observed variables) to the smaller intrinsic dimensionality of feature space (independent variables), which are needed to describe the data economically. Figure 9 shows how the face can be represented by a small number of features. PCA calculates the Eigenvectors of the covariance matrix, and projects the original data onto a lower dimensional feature space, which are defined by Eigenvectors with large Eigenvalues. PCA has been used in face representation and recognition, where the Eigenvectors calculated are referred to as Eigenfaces (as shown in Figure 10).
  An image may also be considering the vector of dimension M×N, so that a typical image of size 4 × 4 becomes a vector of dimension 16. Let the training set of images be $\left\{X_{1},X_{2},\;X_{3}\ldots \;X_{N}\right\}$. The average face of the set is defined by the following:
  (9) $$\bar{X}=\frac{1}{N}\overset{N}{\underset{i=1}{\sum }}X{\;}_{i}.$$
  Calculate the estimate covariance matrix to represent the scatter degree of all feature vectors related to the average vector. The covariance matrix Q is defined by the following:
  (10) $$Q=\frac{1}{N}\overset{N}{\underset{i=1}{\sum }}\left(\bar{X}-X_{i}\right){\left(\bar{X}-X_{i}\right)}^{T}.$$
  The Eigenvectors and corresponding Eigen-values are computed using
  (11) $$CV=\lambda V,\;\left(V\epsilon R_{n},\;V\neq 0\right),$$
  where V is the set of eigenvectors matrix Q associated with its eigenvalue λ. Project all the training images of $i_{th}$ person to the corresponding Eigen-subspace:
  (12) $$y_{k}^{i}=w^{T}\;(x_{i}),\;(i=1,\;2,\;3\;\ldots \;N),$$
  where the $y_{k}^{i}$ are the projections of x and are called the principal components, also known as eigenfaces. The face images are represented as a linear combination of these vectors’ “principal components”. In order to extract facial features, PCA and LDA are two different feature extraction algorithms that are used. Wavelet fusion and neural networks are applied to classify facial features. The ORL database is used for evaluation. Figure 10 shows the first five Eigenfaces constructed from the ORL database [63].
- Fisherface and linear discriminative analysis (LDA) [64,65]: The Fisherface method is based on the same principle of similarity as the Eigenfaces method. The objective of this method is to reduce the high dimensional image space based on the linear discriminant analysis (LDA) technique instead of the PCA technique. The LDA technique is commonly used for dimensionality reduction and face recognition [66]. PCA is an unsupervised technique, while LDA is a supervised learning technique and uses the data information. For all samples of all classes, the within-class scatter matrix $S_{W}$ and the between-class scatter matrix $S_{B}$ are defined as follows:
  (13) $$S_{B}=\sum_{I=1}^{C}M_{i}\left(x_{i}-\mu \right){\left(x_{i}-\mu \right)}^{T},$$
  (14) $$S_{w}=\sum_{I=1}^{C}\underset{x_{k}\epsilon X_{i}}{\sum }M_{i}\left(x_{k}-\mu \right){\left(x_{k}-\mu \right)}^{T},$$
  where μ is the mean vector of samples belonging to class i, $X_{i}$ represents the set of samples belonging to class i with $x_{k}$ being the number image of that class, c is the number of distinct classes, and $M_{i}$ is the number of training samples in class i. $S_{B}$ describes the scatter of features around the overall mean for all face classes and $S_{w}$ describes the scatter of features around the mean of each face class. The goal is to maximize the ratio $det|S_{B}\left|/det\right|S_{w}$|, in other words, minimizing $S_{w}$ while maximiz $\mathrm{ing}\;S_{B}$. Figure 11 shows the first five Eigenfaces and Fisherfaces obtained from the ORL database [63].
- Independent component analysis (ICA) [35]: The ICA technique is used for the calculation of the basic vectors of a given space. The goal of this technique is to perform a linear transformation in order to reduce the statistical dependence between the different basic vectors, which allows the analysis of independent components. It is determined that they are not orthogonal to each other. In addition, the acquisition of images from different sources is sought in uncorrelated variables, which makes it possible to obtain greater efficiency, because ICA acquires images within statistically independent variables.
- Improvements of the PCA, LDA, and ICA techniques: To improve the linear subspace techniques, many types of research are developed. Z. Cui et al. [67] proposed a new spatial face region descriptor (SFRD) method to extract the face region, and to deal with noise variation. This method is described as follows: divide each face image in many spatial regions, and extract token-frequency (TF) features from each region by sum-pooling the reconstruction coefficients over the patches within each region. Finally, extract the SFRD for face images by applying a variant of the PCA technique called “whitened principal component analysis (WPCA)” to reduce the feature dimension and remove the noise in the leading eigenvectors. Besides, the authors in [68] proposed a variant of the LDA called probabilistic linear discriminant analysis (PLDA) to seek directions in space that have maximum discriminability, and are hence most suitable for both face recognition and frontal face recognition under varying pose.
- Gabor filters: Gabor filters are spatial sinusoids located by a Gaussian window that allows for extracting the features from images by selecting their frequency, orientation, and scale. To enhance the performance under unconstrained environments for face recognition, Gabor filters are transformed according to the shape and pose to extract the feature vectors of face image combined with the PCA in the work of [69]. The PCA is applied to the Gabor features to remove the redundancies and to get the best face images description. Finally, the cosine metric is used to evaluate the similarity.
- Frequency domain analysis [70,71]: Finally, the analysis techniques in the frequency domain offer a representation of the human face as a function of low-frequency components that present high energy. The discrete Fourier transform (DFT), discrete cosine transform (DCT), or discrete wavelet transform (DWT) techniques are independent of the data, and thus do not require training.
- Discrete wavelet transform (DWT): Another linear technique used for face recognition. In the work of [70], the authors used a two-dimensional discrete wavelet transform (2D-DWT) method for face recognition using a new patch strategy. A non-uniform patch strategy for the top-level’s low-frequency sub-band is proposed by using an integral projection technique for two top-level high-frequency sub-bands of 2D-DWT based on the average image of all training samples. This patch strategy is better for retaining the integrity of local information, and is more suitable to reflect the structure feature of the face image. When constructing the patching strategy using the testing and training samples, the decision is performed using the neighbor classifier. Many databases are used to evaluate this method, including Labeled Faces in Wild (LFW), Extended Yale B, Face Recognition Technology (FERET), and AR.
- Discrete cosine transform (DCT) [71] can be used for global and local face recognition systems. DCT is a transformation that represents a finite sequence of data as the sum of a series of cosine functions oscillating at different frequencies. This technique is widely used in face recognition systems [71], from audio and image compression to spectral methods for the numerical resolution of differential equations. The required steps to implement the DCT technique are presented as follows.

Owing to their limitations in managing the linearity in face recognition, the subspace or holistic techniques are not appropriate to represent the exact details of geometric varieties of the face images. Linear techniques offer a faithful description of face images when the data structures are linear. However, when the face images data structures are non-linear, many types of research use a function named “kernel” to construct a large space where the problem becomes linear. The required steps to implement the DCT technique are presented as Algorithm 1.
**Algorithm 1.** DCT Algorithm*1.* The input image is N by M;*2.* f(i,j) is the intensity of the pixel in row i and column j;*3.* *F(u,v) is the DCT coefficient in row u and column v of the DCT matrix:*$$F\left(u,v\right)=\frac{2C\left(u\right)C\left(v\right)}{N}\overset{N}{\underset{i=1}{\sum }}\overset{N}{\underset{j=1}{\sum }}f\left(i,j\right)cos\left(\frac{\left(2i-1\right)\left(u-1\right)\pi }{2N}\right)cos\left(\frac{\left(2j-1\right)\left(v-1\right)\pi }{2N}\right)$$$$=\frac{2C\left(u\right)C\left(v\right)}{N}\overset{N-1}{\underset{i=0}{\sum }}\overset{N-1}{\underset{j=0}{\sum }}f\left(i,j\right)cos\left(\frac{\left(2i+1\right)u\pi }{2N}\right)cos\left(\frac{\left(2j+1\right)v\pi }{2N}\right)$$*where*0≤i, v≤N−1*and*$C\left(n\right)=\{\begin{array}{cc} \frac{1}{\sqrt{2}} & \left(n=0\right)\\ 1 & \left(n\neq 0\right) \end{array}$*4.* For most images, much of the signal energy lies at low frequencies; these appear in the upper left corner of the DCT.*5.* Compression is achieved since the lower right values represent higher frequencies, and are often small - small enough to be neglected with little visible distortion.*6.* The DCT input is an 8 by 8 array of integers. This array contains each pixel’s grayscale level;*7.* 8-bit pixels have levels from 0 to 255.

### 4.2. Nonlinear Techniques

- Kernel PCA (KPCA) [28]: is an improved method of PCA, which uses kernel method techniques. KPCA computes the Eigenfaces or the Eigenvectors of the kernel matrix, while PCA computes the covariance matrix. In addition, KPCA is a representation of the PCA technique on the high-dimensional feature space mapped by the associated kernel function. Three significant steps of the KPCA algorithm are used to calculates the function of the kernel matrix K of distribution consisting of n data points $x_{i}\in R^{d}$, after which the data points are mapped into a high-dimensional feature space F, as shown in Algorithm 2.
**Algorithm 2.** Kernel PCA Algorithm*Step 1: Determine the dot product of the matrix*K*using kernel function:*$K_{ij}=k\left(x_{i},x_{j}\right)$.*Step 2: Calculate the Eigenvectors from the resultant matrix*K*and normalize with the function:*γk(αkαk)=1.*Step 3: Calculate the test point projection on to Eigenvectors*Vk*using kernel function:*$kPCk\left(x\right)=\left(Vk\varphi \left(x\right)\right)=\sum_{i}^{m}\alpha k_{i}\;k\left(x_{i},x\right)$
  The performance of the KPCA technique depends on the choice of the kernel matrix K. The Gaussian or polynomial kernel are linear typically-used kernels. KPCA has been successfully used for novelty detection [72] or for speech recognition [62].
- Kernel linear discriminant analysis (KDA) [73]: the KLDA technique is a kernel extension of the linear LDA technique, in the same kernel extension of PCA. Arashloo et al. [73] proposed a nonlinear binary class-specific kernel discriminant analysis classifier (CS-KDA) based on the spectral regression kernel discriminant analysis. Other nonlinear techniques have also been used in the context of facial recognition:
- Gabor-KLDA [74].
- Evolutionary weighted principal component analysis (EWPCA) [75].
- Kernelized maximum average margin criterion (KMAMC), SVM, and kernel Fisher discriminant analysis (KFD) [76].
- Wavelet transform (WT), radon transform (RT), and cellular neural networks (CNN) [77].
- Joint transform correlator-based two-layer neural network [78].
- Kernel Fisher discriminant analysis (KFD) and KPCA [79].
- Locally linear embedding (LLE) and LDA [80].
- Nonlinear locality preserving with deep networks [81].
- Nonlinear DCT and kernel discriminative common vector (KDCV) [82].

### 4.3. Summary of Holistic Approaches

Table 2 summarizes the different subspace techniques discussed in this section, which are introduced to reduce the dimensionality and the complexity of the detection or recognition steps. Linear and non-linear techniques offer robust recognition under different lighting conditions and facial expressions. Although these techniques (linear and non-linear) allow a better reduction in dimensionality and improve the recognition rate, they are not invariant to translations and rotations compared with local techniques.

## 5. Hybrid Approach

### 5.1. Technique Presentation

The hybrid approaches are based on local and subspace features in order to use the benefits of both subspace and local techniques, which have the potential to offer better performance for face recognition systems.

**Figure 12:**
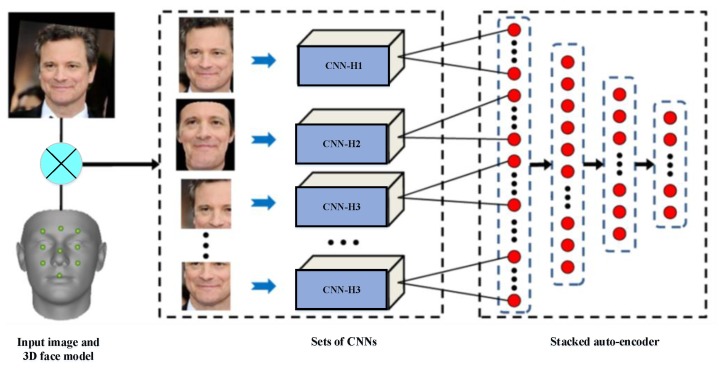
Flowchart of the proposed multimodal deep face representation (MM-DFR) technique [95]. CNN, convolutional neural network.

**Figure 13:**
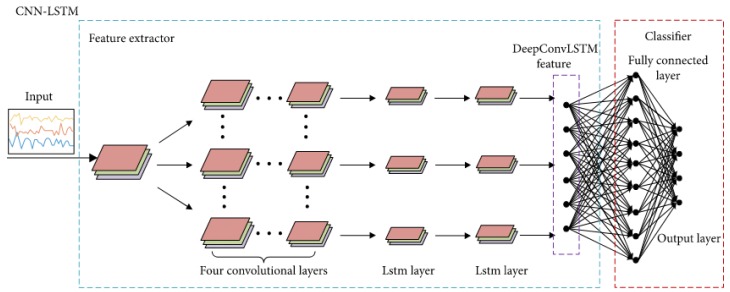
The proposed CNN–LSTM–ELM [103].

- Gabor wavelet and linear discriminant analysis (GW-LDA) [91]: Fathima et al. [91] proposed a hybrid approach combining Gabor wavelet and linear discriminant analysis (HGWLDA) for face recognition. The grayscale face image is approximated and reduced in dimension. The authors have convolved the grayscale face image with a bank of Gabor filters with varying orientations and scales. After that, a subspace technique 2D-LDA is used to maximize the inter-class space and reduce the intra-class space. To classify and recognize the test face image, the k-nearest neighbour (k-NN) classifier is used. The recognition task is done by comparing the test face image feature with each of the training set features. The experimental results show the robustness of this approach in different lighting conditions.
- Over-complete LBP (OCLBP), LDA, and within class covariance normalization (WCCN): Barkan et al. [92] proposed a new representation of face image based over-complete LBP (OCLBP). This representation is a multi-scale modified version of the LBP technique. The LDA technique is performed to reduce the high dimensionality representations. Finally, the within class covariance normalization (WCCN) is the metric learning technique used for face recognition.
- Advanced correlation filters and Walsh LBP (WLBP): Juefei et al. [93] implemented a single-sample periocular-based alignment-robust face recognition technique based on high-dimensional Walsh LBP (WLBP). This technique utilizes only one sample per subject class and generates new face images under a wide range of 3D rotations using the 3D generic elastic model, which is both accurate and computationally inexpensive. The LFW database is used for evaluation, and the proposed method outperformed the state-of-the-art algorithms under four evaluation protocols with a high accuracy of 89.69%.
- Multi-sub-region-based correlation filter bank (MS-CFB): Yan et al. [94] propose an effective feature extraction technique for robust face recognition, named multi-sub-region-based correlation filter bank (MS-CFB). MS-CFB extracts the local features independently for each face sub-region. After that, the different face sub-regions are concatenated to give optimal overall correlation outputs. This technique reduces the complexity, achieves higher recognition rates, and provides a better feature representation for recognition compared with several state-of-the-art techniques on various public face databases.
- SIFT features, Fisher vectors, and PCA: Simonyan et al. [64] have developed a novel method for face recognition based on the SIFT descriptor and Fisher vectors. The authors propose a discriminative dimensionality reduction owing to the high dimensionality of the Fisher vectors. After that, these vectors are projected into a low dimensional subspace with a linear projection. The objective of this methodology is to describe the image based on dense SIFT features and Fisher vectors encoding to achieve high performance on the challenging LFW dataset in both restricted and unrestricted settings.
- CNNs and stacked auto-encoder (SAE) techniques: Ding et al. [95] proposed multimodal deep face representation (MM-DFR) framework based on convolutional neural networks (CNNs) technique from the original holistic face image, rendered frontal face by 3D face model (stand for holistic facial features and local facial features, respectively), and uniformly sampled image patches. The proposed MM-DFR framework has two steps: a CNNs technique is used to extract the features and a three-layer stacked auto-encoder (SAE) technique is employed to compress the high-dimensional deep feature into a compact face signature. The LFW database is used to evaluate the identification performance of MM-DFR. The flowchart of the proposed MM-DFR framework is shown in Figure 12.
- PCA and ANFIS: Sharma et al. [96] propose an efficient pose-invariant face recognition system based on PCA technique and ANFIS classifier. The PCA technique is employed to extract the features of an image, and the ANFIS classifier is developed for identification under a variety of pose conditions. The performance of the proposed system based on PCA–ANFIS is better than ICA–ANFIS and LDA–ANFIS for the face recognition task. The ORL database is used for evaluation.
- DCT and PCA: Ojala et al. [97] develop a fast face recognition system based on DCT and PCA techniques. Genetic algorithm (GA) technique is used to extract facial features, which allows to remove irrelevant features and reduces the number of features. In addition, the DCT–PCA technique is used to extract the features and reduce the dimensionality. The minimum Euclidian distance (ED) as a measurement is used for the decision. Various face databases are used to demonstrate the effectiveness of this system.
- PCA, SIFT, and iterative closest point (ICP): Mian et al. [98] present a multimodal (2D and 3D) face recognition system based on hybrid matching to achieve efficiency and robustness to facial expressions. The Hotelling transform is performed to automatically correct the pose of a 3D face using its texture. After that, in order to form a rejection classifier, a novel 3D spherical face representation (SFR) in conjunction with the SIFT descriptor is used, which provide efficient recognition in the case of large galleries by eliminating a large number of candidates’ faces. A modified iterative closest point (ICP) algorithm is used for the decision. This system is less sensitive and robust to facial expressions, which achieved a 98.6% verification rate and 96.1% identification rate on the complete FRGC v2 database.
- PCA, local Gabor binary pattern histogram sequence (LGBPHS), and GABOR wavelets: Cho et al. [99] proposed a computationally efficient hybrid face recognition system that employs both holistic and local features. The PCA technique is used to reduce the dimensionality. After that, the local Gabor binary pattern histogram sequence (LGBPHS) technique is employed to realize the recognition stage, which proposed to reduce the complexity caused by the Gabor filters. The experimental results show a better recognition rate compared with the PCA and Gabor wavelet techniques under illumination variations. The Extended Yale Face Database B is used to demonstrate the effectiveness of this system.
- PCA and Fisher linear discriminant (FLD) [100,101]: Sing et al. [101] propose a novel hybrid technique for face representation and recognition, which exploits both local and subspace features. In order to extract the local features, the whole image is divided into a sub-regions, while the global features are extracted directly from the whole image. After that, PCA and Fisher linear discriminant (FLD) techniques are introduced on the fused feature vector to reduce the dimensionality. The CMU-PIE, FERET, and AR face databases are used for the evaluation.
- SPCA–KNN [102]: Kamencay et al. [102] develop a new face recognition method based on SIFT features, as well as PCA and KNN techniques. The Hessian–Laplace detector along with SPCA descriptor is performed to extract the local features. SPCA is introduced to identify the human face. KNN classifier is introduced to identify the closest human faces from the trained features. The results of the experiment have a recognition rate of 92% for the unsegmented ESSEX database and 96% for the segmented database (700 training images).
- Convolution operations, LSTM recurrent units, and ELM classifier [103]: Sun et al. [103] propose a hybrid deep structure called CNN–LSTM–ELM in order to achieve sequential human activity recognition (HAR). Their proposed CNN–LSTM–ELM structure is evaluated using the OPPORTUNITY dataset, which contains 46,495 training samples and 9894 testing samples, and each sample is a sequence. The model training and testing runs on a GPU with 1536 cores, 1050 MHz clock speed, and 8 GB RAM. The flowchart of the proposed CNN–LSTM–ELM structure is shown in Figure 13 [103].

### 5.2. Summary of Hybrid Approaches

Table 3 summarizes the hybrid approaches that we presented in this section. Various techniques are introduced to improve the performance and the accuracy of recognition systems. The combination between the local approaches and the subspace approach provides robust recognition and reduction of dimensionality under different illumination conditions and facial expressions. Furthermore, these technologies are presented to be sensitive to noise, and invariant to translations and rotations.

## 6. Assessment of Face Recognition Approaches

In the last step of recognition, the face extracted from the background during the face detection step is compared with known faces stored in a specific database. To make the decision, several techniques of comparison are used. This section describes the most common techniques used to make the decision and comparison.

### 6.1. Measures of Similarity or Distances

- Peak-to-correlation energy (PCE) or peak-to-sidelobe ratio (PSR) [18]: The PCE was introduced in (8).
- Euclidean distance [54]: The Euclidean distance is one of the most basic measures used to compute the direct distance between two points in a plane. If we have two points P1 and P2, with the coordinates (x1, y1) and (x2, y2), respectively, the calculation of the Euclidean distance between them would be as follows:
  (15) $$d_{E}\left(P1,\;P2\;\right)=\sqrt{{\left(x2-x1\right)}^{2}+{\left(y2-y1\right)}^{2}}.$$
  In general, the Euclidean distance between two points P=(1, p2, …, pn) and Q=(q1, q2,… , qn) in the n-dimensional space would be defined by the following:
  (16) $$d_{E}\left(P,Q\right)=\sqrt{\sum_{i}^{n}{\left(pi-qi\right)}^{2}}.$$
- Bhattacharyya distance [104,105]: The Bhattacharyya distance is a statistical measure that quantifies the similarity between two discrete or continuous probability distributions. This distance is particularly known for its low processing time and its low sensitivity to noise. For the probability distributions *p* and *q* defined on the same domain, the distance of Bhattacharyya is defined as follows:
  (17) $$D_{B}\left(p,\;q\right)=-ln\left(BC\left(p,\;q\right)\right),$$
  (18) $$BC\left(p,\;q\right)=\sum_{x\in X}\sqrt{p\left(x\right)q\left(x\right)}\;\left(a\right);\;BC\left(p,\;q\right)=\int \sqrt{p\left(x\right)q\left(x\right)dx}\;\left(b\right),$$
  where BC is the Bhattacharyya coefficient, defined as Equation (18a) for discrete probability distributions and as Equation (18b) for continuous probability distributions. In both cases, 0 ≤ *BC* ≤ 1 and 0 ≤ *DB* ≤ ∞. In its simplest formulation, the Bhattacharyya distance between two classes that follow a normal distribution can be calculated from a mean (μ) and the variance ($\sigma^{2}$):
  (19) $$DB\left(p,\;q\right)=\frac{1}{4}ln\left(\frac{1}{4}\left(\frac{\sigma_{p}^{2}}{\sigma_{q}^{2}}+\frac{\sigma_{q}^{2}}{\sigma_{p}^{2}}+2\right)\right)+\frac{1}{4}\left(\frac{\left(\mu_{p}-\mu_{q}\right)}{\sigma_{q}^{2}+\sigma_{p}^{2}}\right).$$
- Chi-squared distance [106]: The Chi-squared $\left(X^{2}\right)$ distance was weighted by the value of the samples, which allows knowing the same relevance for sample differences with few occurrences as those with multiple occurrences. To compare two histograms $S_{1}=\left(u_{1},\ldots \;\ldots \;\ldots .u_{m}\right)$ and $S_{2}=\left(w_{1},\ldots \;\ldots \;\ldots .w_{m}\right)$, the Chi-squared $\left(X^{2}\right)$ distance can be defined as follows:
  (20) $$\left(X^{2}\right)=D\left(S_{1},S_{2}\right)=\frac{1}{2}\overset{m}{\underset{i=1}{\sum }}\frac{{\left(u_{i}-w_{i}\right)}^{2}}{u_{i}+w_{i}}.$$

### 6.2. Classifiers

There are many face classification techniques in the literature that allow to select, from a few examples, the group or class to which the objects belong. Some of them are based on statistics, such as the Bayesian classifier and correlation [18], and so on, and others based on the regions that generate the different classes in the decision space, such as K-means [9], CNN [103], artificial neural networks (ANNs) [37], support vector machines (SVMs) [26,107], k-nearest neighbors (K-NNs), decision trees (DTs), and so on.

**Figure 14:**
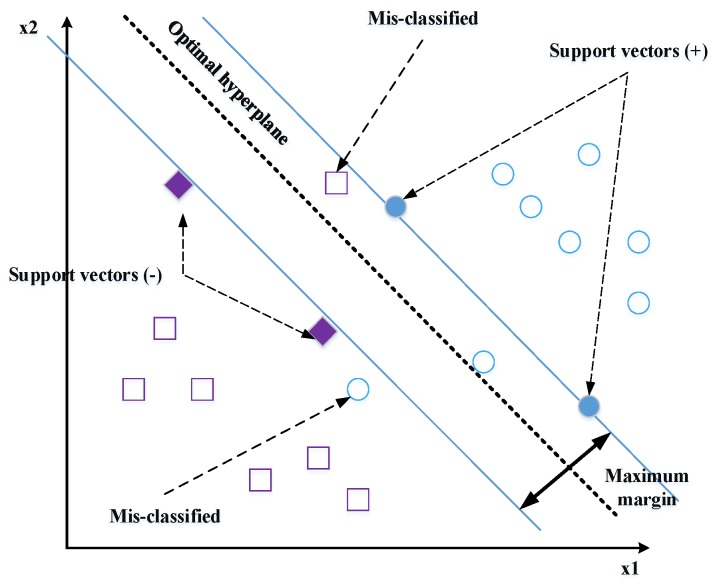
Optimal hyperplane, support vectors, and maximum margin.

**Figure 15:**
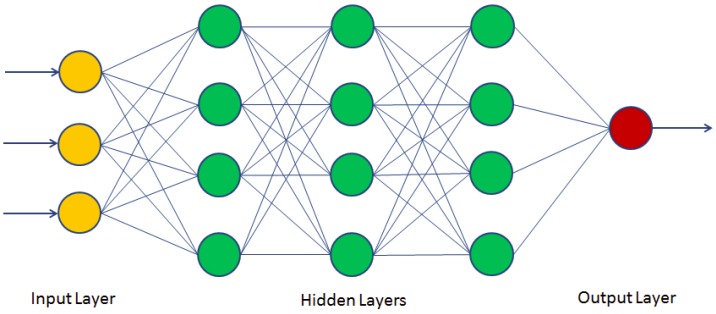
Artificial neural network.

- Support vector machines (SVMs) [13,26]: The feature vectors extracted by any descriptor are classified by linear or nonlinear SVM. The SVM classifier may realize the separation of the classes with an optimal hyperplane. To determine the last, only the closest points of the total learning set should be used; these points are called support vectors (Figure 14).
  There is an infinite number of hyperplanes capable of perfectly separating two classes, which implies to select a hyperplane that maximizes the minimal distance between the learning examples and the learning hyperplane (i.e., the distance between the support vectors and the hyperplane). This distance is called “margin”. The SVM classifier is used to calculate the optimal hyperplane that categorizes a set of labels training data in the correct class. The optimal hyperplane is solved as follows:
  (21) $$D=\left\{\left(x_{i},y_{i}\right)|x_{i}\in R^{n},\;y_{i}\in \left\{-1,1\right\},\;i=1\ldots \ldots l\right\}.$$
  Given that $x_{i}$ are the training features vectors and $y_{i}$ are the corresponding set of l (1 or −1) labels. An SVM tries to find a hyperplane to distinguish the samples with the smallest errors. The classification function is obtained by calculating the distance between the input vector and the hyperplane.
  (22) $$wx_{i}-b=C_{f},$$
  where w and b are the parameters of the model. Shen et al. [108] proposed the Gabor filter to extract the face features and applied the SVM for classification. The proposed FaceNet method achieves a good record accuracy of 99.63% and 95.12% using the LFW YouTube Faces DB datasets, respectively.
- k-nearest neighbor (k-NN) [17,91]: k-NN is an indolent algorithm because, in training, it saves little information, and thus does not build models of difference, for example, decision trees.
- K-means [9,109]: It is called K-means because it represents each of the groups by the average (or weighted average) of its points, called the centroid. In the K-means algorithm, it is necessary to specify a priori the number of clusters k that one wishes to form in order to start the process.
- Deep learning (DL): An automatic learning technique that uses neural network architectures. The term “deep” refers to the number of hidden layers in the neural network. While conventional neural networks have one layer, deep neural networks (DNN) contain several layers, as presented in Figure 15.

Various variants of neural networks have been developed in the last years, such as convolutional neural networks (CNN) [14,110] and recurrent neural networks (RNN) [111], which very effective for image detection and recognition tasks. CNNs are a very successful deep model and are used today in many applications [112]. From a structural point of view, CNNs are made up of three different types of layers: convolution layers, pooling layers, and fully-connected layers.

1. *Convolutional layer*: sometimes called the feature extractor layer because features of the image are extracted within this layer. Convolution preserves the spatial relationship between pixels by learning image features using small squares of the input image. The input image is convoluted by employing a set of learnable neurons. This produces a feature map or activation map in the output image, after which the feature maps are fed as input data to the next convolutional layer. The convolutional layer also contains rectified linear unit (ReLU) activation to convert all negative value to zero. This makes it very computationally efficient, as few neurons are activated each time.
2. *Pooling layer:* used to reduce dimensions, with the aim of reducing processing times by retaining the most important information after convolution. This layer basically reduces the number of parameters and computation in the network, controlling over fitting by progressively reducing the spatial size of the network. There are two operations in this layer: average pooling and maximum pooling:
  - Average-pooling takes all the elements of the sub-matrix, calculates their average, and stores the value in the output matrix.
  - Max-pooling searches for the highest value found in the sub-matrix and saves it in the output matrix.
3. *Fully-connected layer*: in this layer, the neurons have a complete connection to all the activations from the previous layers. It connects neurons in one layer to neurons in another layer. It is used to classify images between different categories by training.

Wen et al. [113] introduce a new supervision signal, called center loss, for the face recognition task in order to improve the discriminative power of the deeply learned features. Specifically, the proposed center loss function is trainable and easy to optimize in the CNNs. Several important face recognition benchmarks are used for evaluation including LFW, YTF, and MegaFace Challenge. Passalis and Tefas [114] propose a supervised codebook learning method for the bag-of-features representation able to learn face retrieval-oriented codebooks. This allows using significantly smaller codebooks enhancing both the retrieval time and storage requirements. Liu et al. [115] and Amato et al. [116] propose a deep face recognition technique under open-set protocol based on the CNN technique. A face dataset composed of 39,037 faces images belonging to 42 different identities is used to perform the experiments. Taigman et al. [117] present a system (DeepFace) able to outperform existing systems with only very minimal adaptation. It is trained on a large dataset of faces acquired from a population vastly different than the one used to construct the evaluation benchmarks. This technique achieves an accuracy of 97.35% on the LFW. Ma et al. [118] introduce a robust local binary pattern (LBP) guiding pooling (G-RLBP) mechanism to improve the recognition rates of the CNN models, which can successfully lower the noise impact. Koo et al. [119] propose a multimodal human recognition method that uses both the face and body and is based on a deep CNN. Cho et al. [120] propose a nighttime face detection method based on CNN technique for visible-light images. Koshy and Mahmood [121] develop deep architectures for face liveness detection that uses a combination of texture analysis and a CNN technique to classify the captured image as real or fake. Elmahmudi and Ugail [122] present the performance of machine learning for face recognition using partial faces and other manipulations of the face such as rotation and zooming, which we use as training and recognition cues. The experimental results on the tasks of face verification and face identification show that the model obtained by the proposed DNN training framework achieves 97.3% accuracy on the LFW database with low training complexity. Seibold et al. [123] proposed a morphing attack detection method based on DNNs. A fully automatic face image morphing pipeline with exchangeable components was used to generate morphing attacks, train neural networks based on these data, and analyze their accuracy. Yim et al. [124] propose a new deep architecture based on a novel type of multitask learning, which can achieve superior performance in rotating to a target-pose face image from an arbitrary pose and illumination image while preserving identity. Nguyen et al. [111] propose a new approach for detecting presentation attack face images to enhance the security level of a face recognition system. The objective of this study was the use of a very deep stacked CNN–RNN network to learn the discrimination features from a sequence of face images. Finally, Bajrami et al. [125] present experiment results with LDA and DNN for face recognition, while their efficiency and performance are tested on the LFW dataset. The experimental results show that the DNN method achieves better recognition accuracy, and the recognition time is much faster than that of the LDA method in large-scale datasets.

### 6.3. Databases Used

The most commonly used databases for face recognition systems under different conditions are Pointing Head Pose Image Database (PHPID) [126], Labeled Faces in Wild (LFW) [127], FERET [15,16], ORL, and Yale. The last are used for face recognition systems under different conditions, which provide information for supervised and unsupervised learning. Supervised learning is based on two training modules: image unrestricted training setting and image restricted training setting. For the first model, only “same” or “not same” binary labels are used in the training splits. For the second model, the identities of the person in each pair are provided in the training splits.

- LFW (Labeled Faces in the Wild) database was created in October 2007. It contains 13,333 images of 5749 subjects, with 1680 subjects with at least two images and the rest with a single image. These face images were taken on the Internet, pre-processed, and localized by the Viola–Jones detector with a resolution of 250 × 250 pixels. Most of them are in color, although there are also some in grayscale and presented in JPG format and organized by folders.
- FERET (Face Recognition Technology) database was created in 15 sessions in a semi-controlled environment between August 1993 and July 1996. It contains 1564 sets of images, with a total of 14,126 images. The duplicate series belong to subjects already present in the series of individual images, which were generally captured one day apart. Some images taken from the same subject vary overtime for a few years and can be used to treat facial changes that appear over time. The images have a depth of 24 bits, RGB, so they are color images, with a resolution of 512 × 768 pixels.
- AR face database was created by Aleix Martínez and Robert Benavente in the computer vision center (CVC) of the Autonomous University of Barcelona in June 1998. It contains more than 4000 images of 126 subjects, including 70 men and 56 women. They were taken at the CVC under a controlled environment. The images were taken frontally to the subjects, with different facial expressions and three different lighting conditions, as well as several accessories: scarves, glasses, or sunglasses. Two imaging sessions were performed with the same subjects, 14 days apart. These images are a resolution of 576 × 768 pixels and a depth of 24 bits, under the RGB RAW format.
- ORL Database of Faces was performed between April 1992 and April 1994 at the AT & T laboratory in Cambridge. It consists of a total of 10 images per subject, out of a total of 40 images. For some subjects, the images were taken at different times, with varying illumination and facial expressions: eyes open/closed, smiling/without a smile, as well as with or without glasses. The images were taken under a black homogeneous background, in a vertical position and frontally to the subject, with some small rotation. These are images with a resolution of 92 × 112 pixels in grayscale.
- Extended Yale Face B database contains 16,128 images of 640 × 480 grayscale of 28 individuals under 9 poses and 64 different lighting conditions. It also includes a set of images made with the face of individuals only.
- Pointing Head Pose Image Database (PHPID) is one of the most widely used for face recognition. It contains 2790 monocular face images of 15 persons with tilt angles from −90° to +90° and variations of pan. Every person has two series of 93 different poses (93 images). The face images were taken under different skin color and with or without glasses.

### 6.4. Comparison between Holistic, Local, and Hybrid Techniques

In this section, we present some advantages and disadvantages of holistic, local, and hybrid approaches to identifying faces during the last 20 years. DL approaches can be considered as a statistical approach (holistic method), because the training procedure scheme usually searches for statistical structures in the input patterns. Table 4 presents a brief summary of the three approaches.

## 7. Discussion about Future Directions and Conclusions

### 7.1. Discussion

In the past decade, the face recognition system has become one of the most important biometric authentication methods. Many techniques are used to develop many face recognition systems based on facial information. Generally, the existing techniques can be classified into three approaches, depending on the type of desired features.

- Local approaches: use features in which the face described partially. For example, some system could consist of extracting local features such as the eyes, mouth, and nose. The features’ values are calculated from the lines or points that can be represented on the face image for the recognition step.
- Holistic approaches: use features that globally describe the complete face as a model, including the background (although it is desirable to occupy the smallest possible surface).
- Hybrid approaches: combine local and holistic approaches.

In particular, recognition methods performed on static images produce good results under different lighting and expression conditions. However, in most cases, only the face images are processed at the same size and scale. Many methods require numerous training images, which limits their use for real-time systems, where the response time is an important aspect.

The main purpose of techniques such as HOG, LBP, Gabor filters, BRIEF, SURF, and SIFT is to discover distinctive features, which can be divided into two parts: (1) local appearance-based techniques, which are used to extract local features when the face image is divided into small regions (including HOG, LBP, Gabor filters, and correlation filters); and (2) key-points-based techniques, which are used to detect the points of interest in the face image, after which features’ extraction is localized based on these points, including BRIEF, SURF, and SIFT. In the context of face recognition, local techniques only treat certain facial features, which make them very sensitive to facial expressions and occlusions [4,14,37,50,51,52,53]. The relative robustness is the main advantage of these feature-based local techniques. Additionally, they take into account the peculiarity of the face as a natural form to recognize a reduced number of parameters. Another advantage is that they have a high compaction capacity and a high comparison speed. The main disadvantages of these methods are the difficulty of automating the detection of facial features and the fact that the person responsible for the implementation of these systems must make an arbitrary decision on really important points.

Unlike the local approaches, holistic approaches are other methods used for face recognition, which treat the whole face image and do not require extracting face regions or features points (eyes, mouth, noses, and so on). The main function of these approaches is to represent the face image with a matrix of pixels. This matrix is often converted into feature vectors to facilitate their treatment. After that, the feature vectors are applied in a low-dimensional space. In fact, subspace techniques are sensitive to different variations (facial expressions, illumination, and different poses), which make them easy to implement. Many subspace techniques are implemented to represent faces such as Eigenface, Eigenfisher, PCA, and LDA, which can be divided into two categories: linear and non-linear techniques. The main advantage of holistic approaches is that they do not destroy image information by focusing only on regions or points of interest. However, this property represents a disadvantage because it assumes that all the pixels of the image have the same importance. As a result, these techniques are not only computationally expensive, but also require a high degree of correlation between the test and the training images. In addition, these approaches generally ignore local details, which means they are rarely used to identify faces.

Hybrid approaches are based on local and global features to exploit the benefits of both techniques. These approaches combine the two approaches described above into a single system to improve the performance and accuracy of recognition. The choice of the required method to be used must take into account the application in which it was applied. For example, in the face recognition systems that use very small images, methods based on local features are a bad choice. Another consideration in the algorithm selection process is the number of training examples needed. Finally, we can remember that the tendency is to develop hybrid methods that combine the advantages of local and holistic approaches, but these methods are very complex and require more processing time.

A notable limitation that we found in all the publications reviewed is methodological: despite that the 2D facial recognition has reached a significant level of maturity and a high success rate, it is not surprising that it continues to be one of the most active research areas in computer vision. Considering the results published to date, in the opinion of these authors, three particularly promising techniques for further development of this area stand out: (i) the development of 3D face recognition methods; (ii) the use of multimodal fusion methods of complementary data types, in particular those based on visible and infrared images; and (iii) the use of DL methods.

1. Three-dimensional face recognition: In 2D image-based techniques, some features are lost owing to the 3D structure of the face. Lighting and pose variations are two major unresolved problems of 2D face recognition. Recently, 3D facial recognition for facial recognition has been widely studied by the scientific community to overcome unresolved problems in 2D facial recognition and to achieve significantly higher accuracy by measuring geometry of rigid features on the face. For this reason, several recent systems based on 3D data have been developed [3,93,95,128,129].
2. Multimodal facial recognition: sensors have been developed in recent years with a proven ability to acquire not only two-dimensional texture information, but also facial shape, that is, three-dimensional information. For this reason, some recent studies have merged the two types of 2D and 3D information to take advantage of each of them and obtain a hybrid system that improves the recognition as the only modality [98].
3. Deep learning (DL): a very broad concept, which means that it has no exact definition, but studies [14,110,111,112,113,121,130,131] agree that DL includes a set of algorithms that attempt to model high level abstractions, by modeling multiple processing layers. This field of research began in the 1980s and is a branch of automatic learning where algorithms are used in the formation of deep neural networks (DNN) to achieve greater accuracy than other classical techniques. In recent progress, a point has been reached where DL performs better than people in some tasks, for example, to recognize objects in images.

Finally, researchers have gone further by using multimodal and DL facial recognition systems.

### 7.2. Conclusions

Face recognition system is a popular study task in the field of image processing and computer vision, owing to its potentially enormous application as well as its theoretical value. This system is widely deployed in many real-world applications such as security, surveillance, homeland security, access control, image search, human-machine, and entertainment. However, these applications pose different challenges such as lighting conditions and facial expressions. This paper highlights the recent research on the 2D or 3D face recognition system, focusing mainly on approaches based on local, holistic (subspace), and hybrid features. A comparative study between these approaches in terms of processing time, complexity, discrimination, and robustness was carried out. We can conclude that local feature techniques are the best choice concerning discrimination, rotation, translation, complexity, and accuracy. We hope that this survey paper will further encourage researchers in this field to participate and pay more attention to the use of local techniques for face recognition systems.

## Figures and Tables

**Table 1:** Summary of local approaches. SIFT, scale-invariant feature transform; SURF, scale-invariant feature transform; BRIEF, binary robust independent elementary features; LBP, local binary pattern; HOG, histogram of oriented gradients; LPQ, local phase quantization; PCA, principal component analysis; LDA, linear discriminant analysis; KPCA, kernel PCA; CNN, convolutional neural network; SVM, support vector machine; PLBP, pyramid of LBP; KNN, k-nearest neighbor; MLBP, multiscale LBP; LTP, local ternary pattern.; PHOG, pyramid HOG; VLC, VanderLugt correlator; LFW, Labeled Faces in the Wild; FERET, Face Recognition Technology; PHPID, Pointing Head Pose Image Database; PCE, peak to correlation energy; POF, phase-only filter; PSR, peak-to-sidelobe ratio.

Author/Technique Used		Database	Matching	Limitation	Advantage	Result
Local Appearance-Based Techniques						
Khoi et al. [20]	LBP	TDF	MAP	Skewness in face image	Robust feature in fontal face	5%
		CF1999				13.03%
		LFW				90.95%
Xi et al. [15]	LBPNet	FERET	Cosine similarity	Complexities of CNN	High recognition accuracy	97.80%
		LFW				94.04%
Khoi et al. [20]	PLBP	TDF	MAP	Skewness in face image	Robust feature in fontal face	5.50%
		CF				9.70%
		LFW				91.97%
Laure et al. [40]	LBP and KNN	LFW	KNN	Illumination conditions	Robust	85.71%
		CMU-PIE				99.26%
Bonnen et al. [42]	MRF and MLBP	AR (Scream)	Cosine similarity	Landmark extraction fails or is not ideal	Robust to changes in facial expression	86.10%
		FERET (Wearing sunglasses)				95%
Ren et al. [43]	Relaxed LTP	CMU-PIE	Chisquare distance	Noise level	Superior performance compared with LBP, LTP	95.75%
		Yale B				98.71%
Hussain et al. [60]	LPQ	FERET/	Cosine similarity	Lot of discriminative information	Robust to illumination variations	99.20%
		LFW				75.30%
Karaaba et al. [44]	HOG and MMD	FERET	MMD/MLPD	Low recognition accuracy	Aligning difficulties	68.59%
		LFW				23.49%
Arigbabu et al. [46]	PHOG and SVM	LFW	SVM	Complexity and time of computation	Head pose variation	88.50%
Leonard et al. [50]	VLC correlator	PHPID	ASPOF	The low number of the reference image used	Robustness to noise	92%
Napoléon et al. [38]	LBP and VLC	YaleB	POF	Illumination	Rotation + Translation	98.40%
		YaleB Extended				95.80%
Heflin et al. [54]	correlation filter	LFW/PHPID	PSR	Some pre-processing steps	More effort on the eye localization stage	39.48%
Zhu et al. [55]	PCA–FCF	CMU-PIE	Correlation filter	Use only linear method	Occlusion-insensitive	96.60%
		FRGC2.0				91.92%
Seo et al. [27]	LARK + PCA	LFW	Cosine similarity	Face detection	Reducing computational complexity	78.90%
Ghorbel et al. [61]	VLC + DoG	FERET	PCE	Low recognition rate	Robustness	81.51%
Ghorbel et al. [61]	uLBP + DoG	FERET	chi-square distance	Robustness	Processing time	93.39%
Ouerhani et al. [18]	VLC	PHPID	PCE	Power	Processing time	77%
**Key-Points-Based Techniques**						
Lenc et al. [56]	SIFT	FERET	a posterior probability	Still far to be perfect	Sufficiently robust on lower quality real data	97.30%
		AR				95.80%
		LFW				98.04%
Du et al. [29]	SURF	LFW	FLANN distance	Processing time	Robustness and distinctiveness	95.60%
Vinay et al. [23]	SURF + SIFT	LFW	FLANN	Processing time	Robust in unconstrained scenarios	78.86%
		Face94	distance			96.67%
Calonder et al. [30]	BRIEF	_	KNN	Low recognition rate	Low processing time	48%

**Table 2:** Subspace approaches. ICA, independent component analysis; DWT, discrete wavelet transform; FFT, fast Fourier transform; DCT, discrete cosine transform.

Author/Techniques Used		Databases	Matching	Limitation	Advantage	Result
		Linear Techniques				
Seo et al. [27]	LARK and PCA	LFW	L2 distance	Detection accuracy	Reducing computational complexity	85.10%
Annalakshmi et al. [35]	ICA and LDA	LFW	Bayesian Classifier	Sensitivity	Good accuracy	88%
Annalakshmi et al. [35]	PCA and LDA	LFW	Bayesian Classifier	Sensitivity	Specificity	59%
Hussain et al. [36]	LQP and Gabor	FERET	Cosine similarity	Lot of discriminative information	Robust to illumination variations	99.2% 75.3%
		LFW				
Gowda et al. [17]	LPQ and LDA	MEPCO	SVM	Computation time	Good accuracy	99.13%
Z. Cui et al. [67]	BoW	AR	ASM	Occlusions	Robust	99.43%
		ORL				99.50%
		FERET				82.30%
Khan et al. [83]	PSO and DWT	CK	Euclidienne distance	Noise	Robust to illumination	98.60%
		MMI				95.50%
		JAFFE				98.80%
Huang et al. [70]	2D-DWT	FERET	KNN	Pose	Frontal or near-frontal facial images	90.63% 97.10%
		LFW				
Perlibakas and Vytautas [69]	PCA and Gabor filter	FERET	Cosine metric	Precision	Pose	87.77%
Hafez et al. [84]	Gabor filter and LDA	ORL	2DNCC	Pose	Good recognition performance	98.33%
		C. YaleB				99.33%
Sufyanu et al. [71]	DCT	ORL	NCC	High memory	Controlled and uncontrolled databases	93.40%
		Yale				
Shanbhag et al. [85]	DWT and BPSO	_ _	_ _	Rotation	Significant reduction in the number of features	88.44%
Ghorbel et al. [61]	Eigenfaces and DoG filter	FERET	Chi-square distance	Processing time	Reduce the representation	84.26%
Zhang et al. [12]	PCA and FFT	YALE	SVM	Complexity	Discrimination	93.42%
Zhang et al. [12]	PCA	YALE	SVM	Recognition rate	Reduce the dimensionality	84.21%
		**Nonlinear Techniques**				
Fan et al. [86]	RKPCA	MNIST ORL	RBF kernel	Complexity	Robust to sparse noises	_
Vinay et al. [87]	ORB and KPCA	ORL	FLANN Matching	Processing time	Robust	87.30%
Vinay et al. [87]	SURF and KPCA	ORL	FLANN Matching	Processing time	Reduce the dimensionality	80.34%
Vinay et al. [87]	SIFT and KPCA	ORL	FLANN Matching	Low recognition rate	Complexity	69.20%
Lu et al. [88]	KPCA and GDA	UMIST face	SVM	High error rate	Excellent performance	48%
Yang et al. [89]	PCA and MSR	HELEN face	ESR	Complexity	Utilizes color, gradient, and regional information	98.00%
Yang et al. [89]	LDA and MSR	FRGC	ESR	Low performances	Utilizes color, gradient, and regional information	90.75%
Ouanan et al. [90]	FDDL	AR	CNN	Occlusion	Orientations, expressions	98.00%
Vankayalapati and Kyamakya [77]	CNN	ORL	_ _	Poses	High recognition rate	95%
Devi et al. [63]	2FNN	ORL	_ _	Complexity	Low error rate	98.5

**Table 3:** Hybrid approaches. GW, Gabor wavelet; OCLBP, over-complete LBP; WCCN, within class covariance normalization; WLBP, Walsh LPB; ICP, iterative closest point; LGBPHS, local Gabor binary pattern histogram sequence; FLD, Fisher linear discriminant; SAE, stacked auto-encoder.

Author/Technique Used		Database	Matching	Limitation	Advantage	Result
Fathima et al. [91]	GW-LDA	AT&T	k-NN	High processing time	Illumination invariant and reduce the dimensionality	88%
		FACES94				94.02%
		MITINDIA				88.12%
Barkan et al., [92]	OCLBP, LDA, and WCCN	LFW	WCCN	_	Reduce the dimensionality	87.85%
Juefei et al. [93]	ACF and WLBP	LFW		Complexity	Pose conditions	89.69%
Simonyan et al. [64]	Fisher + SIFT	LFW	Mahalanobis matrix	Single feature type	Robust	87.47%
Sharma et al. [96]	PCA–ANFIS	ORL	ANFIS	Sensitivity-specificity		96.66%
	ICA–ANFIS		ANFIS		Pose conditions	71.30%
	LDA–ANFIS		ANFIS			68%
Ojala et al. [97]	DCT–PCA	ORL	Euclidian distance	Complexity	Reduce the dimensionality	92.62%
		UMIST				99.40%
		YALE				95.50%
Mian et al. [98]	Hotelling transform, SIFT, and ICP	FRGC	ICP	Processing time	Facial expressions	99.74%
Cho et al. [99]	PCA–LGBPHS	Extended Yale Face	Bhattacharyya distance	Illumination condition	Complexity	95%
	PCA–GABOR Wavelets					
Sing et al. [101]	PCA–FLD	CMU	SVM	Robustness	Pose, illumination, and expression	71.98%
		FERET				94.73%
		AR				68.65%
Kamencay et al. [102]	SPCA-KNN	ESSEX	KNN	Processing time	Expression variation	96.80%
Sun et al. [103]	CNN–LSTM–ELM	OPPORTUNITY	LSTM/ELM	High processing time	Automatically learn feature representations	90.60%
Ding et al. [95]	CNNs and SAE	LFW	_ _	Complexity	High recognition rate	99%

**Table 4:** General performance of face recognition approaches.

Approaches		Databases Used	Advantages	Disadvantages	Performances	Challenges Handled
**Local**	**Local Appearance**	TDF, CF1999, LFW, FERET, CMU-PIE, AR, Yale B, PHPID, YaleB Extended, FRGC2.0, Face94.	Easy to implement, allowing an analysis of images in a difficult environment in real-time [38]. Invariant to size, orientation, and lighting [47,48].	Lack discrimination ability. It is difficult to automatic detect feature in this approach.	High performance in terms of processing time and recognition rate [15,38].	Pose variations [42], various lighting conditions[60], facial expressions [38], and low resolution.
	**Key-Points**		Does not require prior knowledge of the images [56]. Different illumination conditions, scaling, aging effects, facial expressions, face occlusions, and noisy images [57].	More affected by orientation changes or the expression of the face [23].	High processing time [29]. Low recognition rate [30].	Different illumination conditions, facial expressions, aging effects, scaling, face occlusions and noisy images [56].
**Holistic**	**Linear**	LFW, FERET, MEPCO, AR, ORL, CK, MMI, JAFFE, C. Yale B, Yale, MNIST, ORL, UMIST face, HELEN face, FRGC.	When frontal views of faces are used, these techniques provide good performance [35,70]. Recognition is effective and simple. Dimensionality reduction, represent global information [17,27,67,70].	Sensitive to the rotation and the translation of the face images. Can only classify a face that is “known” to the database. Low speed in the face recognition caused by a long feature vector [36].	Processed with larger size features. High processing time [17]. High performance in terms of recognition rate [67].	Different illumination conditions [36,83], scaling, facial expressions.
	**Non-Linear**		Dimensionality reduction [86,87,88]. They are because of supervised classification problems. Automatically detect feature in this approach (CNN and RNN) [63,77,90].	The recognition performance depends on the chosen kernel [88]. More difficult to implement than the local technique. Recognition rate unsatisfying [87,88].	Complexity [88]. Computationally expensive and require a high degree of correlation between the test and training images (SVM, CNN) [88,90].	Different illumination [36,83], poses [70], conditions, scaling, facial expressions.
**Hybrid**		AT&T, FACES94, MITINDIA, LFW, ORL, UMIST, YALE, FRGC, Extended Yale, CMU, FERET, AR, ESSEX.	Provides faster systems and efficient recognition [95].	More difficult to implement. Complex and computational cost [93,95,97].	High recognition rate [95]. High computational complexity [97].	Pose, illumination conditions, and facial expressions [101,102].
